# Pathfast Presepsin Assay for Early Diagnosis of Systemic Inflammatory Response Syndrome in Patients with Nephrolithiasis

**DOI:** 10.1155/2015/792572

**Published:** 2015-02-04

**Authors:** Yan-song Hou, Hua Wang, Hao Chen, Ling-feng Wu, Lin-feng Lu, Yi He

**Affiliations:** Department of Urology, First Affiliated Hospital of Jiaxing Medical College, Jiaxing 314000, China

## Abstract

It is relatively difficult to diagnose bacterial sepsis in nephrolithiasis patients. The aim of the study is to evaluate the diagnostic ability of presepsin in the differential diagnosis including SIRS, infection, or sepsis and to compare its diagnostic value with other markers, mainly as CRP, procalcitonin (PCT), and white blood cell (WBC) in patients of nephrolithiasis presenting with SIRS. 39 patients of nephrolithiasis who were diagnosed as SIRS were prospectively investigated. Plasma presepsin was detected by Pathfast presepsin assay system; CRP and PCT were measured as well. Additionally, 25 nephrolithiasis patients without SIRS were included. At all timing samples, patients were classified as SIRS or non-SIRS group. Median plasma presepsin levels were significantly increased in the SIRS group compared with non-SIRS group (452 pg/mL versus 178 ng/mL, *P* < 0.001), and presepsin was markedly elevated even in the early stage of SIRS (584 pg/mL 6 h, 660 pg/mL 24 h versus 452 pg/mL, *P* < 0.001). According to the receiver-operating characteristic (ROC) analysis, presepsin demonstrated a high diagnostic value compared with either PCT or CRP. In the early stage of SIRS, presepsin remained a highly sensitive (74.7%) and specific (88.4%) diagnostic marker compared with either PCT, CRP, or WBC. Moreover, the areas under the curve (AUCs) of presepsin (84.6%) were also superior to those seen in either PCT (79.6%) or CRP (71.8%). Thus plasma presepsin levels have comparable performance in SIRS for patients with nephrolithiasis.

## 1. Introduction

Nephrolithiasis can be caused by multiple factors and may exist in any part of the urinary system with renal calculus being the most common [1, 2]. Percutaneous nephrolithotomy (PNL) is the main treatment modalities for nephrolithiasis in the present. PNL can have great influences on the body because of trauma, pain, infection, inflammation, and so forth, so stress response may lead to the elevation of inflammatory factors, such as the acute phase C-reactive protein (CRP), cytokines interleukin-1 (IL-1), interleukin-6 (IL-6), interleukin-2 (IL-2), interleukin-10 (IL-10), and TNF-*α* [3, 4]. Systemic inflammatory response syndrome (SIRS) accompanied by an infection is called sepsis, in the field of urology; it is also called urosepsis. Despite of preoperative antibiotic treatment, the sepsis rates have been reported to vary from 0.25 to 1.5% in PNL studies, and in developing country such as china, the incidence of urosepsis is relatively higher and the status of urinary tract infection (UTI) accompanied with nephrolithiasis should be characterized with close concern [5, 6]. Since the early manifestation is usually obscure, sepsis is often ignored in clinic. For many urologists, the lack of standardizations in accurate diagnosis of SIRS caused severe multiorgan dysfunction and even fetal complications [7]. In fact, the criteria for the diagnosis of infection and sepsis in nonnephrolithiasis patients should not be applied to the cohort of nephrolithiasis patients because the inflammatory mediators may alter during the process of PNL, and traditional diagnostic criteria of SIRS and sepsis lack diagnostic accuracy and are sometimes misleading.

Various markers have been studied for diagnosing sepsis. At present, serum levels procalcitonin (PCT) and C-reactive protein (CRP) are the two most common parameters in the diagnosis of SIRS [8, 9]. However, the level of PCT increases as a reflection of the severity of the body's reaction to the traumatic stimuli, so in the absence of signs of infection, diagnostic performance of PCT for infection was low in the cases like trauma and postoperation patients. It is not clear yet whether the increase of CRP in nephrolithiasis patients is related to the presence of septic complications or is an effect of surgical-related trauma itself. White blood cell (WBC), a criterion for sepsis, is routinely performed in almost every patient but it is influenced by many noninfectious factors [10, 11]. So in patients with nephrolithiasis that received PNL, there is a need for reevaluation of inflammation markers for the diagnosis of SIRS.

Soluble cluster of differentiation 14 subtype (sCD14-ST), also called presepsin, is cleaved from the monocyte/macrophage-specific CD14 receptor complex after binding with lipopolysaccharides (LPS) and LPS binding protein (LPB) during systemic infections [12–15]. Aspartate proteases, including cathepsin D, were one of the lysosomal enzymes that are related to the production of presepsin [13]. It is reported that measurement of presepsin concentrations is useful for diagnosis of sepsis and also for monitoring clinical responses to therapeutic interventions [16, 17].

Here, we intend to evaluate the diagnostic ability of presepsin in the differential diagnosis including SIRS, infection, or sepsis and to compare its diagnostic value with other markers, mainly as CRP, PCT, and WBC in patients of nephrolithiasis presenting with SIRS. Diagnosis of SIRS in the nephrolithiasis patients is sometimes not timely; hence a marker that is able to distinguish inflammatory response to infection from other causes of inflammation should be tried in clinical practice and the advantage of using presepsin as a marker is its accuracy, which will be demonstrated in this work [18]. Besides, we have analyzed the data from samples collected during the immediate occurrence of SIRS and the once more 24 h later.

## 2. Materials and Methods

### 2.1. Patients

Between July 2013 and June 2014, all in-patients clinically diagnosed as nephrolithiasis by imaging examination at our department were enrolled in the study. Diagnostic criteria of SIRS were based on clinical manifestations and laboratory findings, according to (American College of Chest Physicians/Society of Critical Care Medicine) ACCP/SCCM guidelines: body temperature >38.5°C or <36°C, heart rate (HR) > 90/min, respiratory rate (RR) > 20/min, or PaCO_2_ < 32 mmHg and leukocyte count >12 × 10^9^/L or <4 × 10^9^/L, levels > 10% of immature neutrophils; then the simultaneous presence of two or more of these criteria clinical/laboratory signs was defined as SIRS [19]. In this study, there were 24 men and 15 women in the nephrolithiasis with SIRS group, and 16 men and 9 women in the non-SIRS group. Blood cultures were taken routinely and urine cultures are taken when necessary. Culture (+) sepsis diagnosis could be made the day culture results were taken but sepsis day was labeled retrospectively as the day blood culture was sampled. At all timing samples, whole criteria were evaluated and situation was classified as SIRS and non-SIRS according to ACCP criteria by the same microbiologist and urologists. To exclude presepsin levels that might have increased due to acute kidney injury (AKI), we have used the diagnostic criteria for AKI which are proposed by the Acute Kidney Injury Network (AKIN) [20]: an abrupt (within 48 h) reduction in kidney function is currently defined as an absolute increase in serum creatinine levels of more than or equal to 26.4 *μ*mol/L, a percentage increase in serum creatinine of more than or equal to 50%. We have also collected blood urea nitrogen (BUN), serum creatinine (SCr), and urinary WBC at the time when SIRS was evident and once more 48 h after therapy. Approval for the study was received in advance from the local ethics committee of our institution.

### 2.2. Samples Collection and Detection

After obtaining informed consent, the serum samples of SIRS patients were obtained immediately when the symptoms occurred before antibiotics intervention. The second and third serum samples were obtained after 6 h and 24 h. Plasma and serum supernatants were obtained after centrifugation of the blood samples for 10 min, following which samples were stored in 0.5 mL aliquots at −80°C in order to avoid multiple freeze-thaw cycles. Plasma presepsin levels were measured by a chemiluminescent enzyme immunoassay (Pathfast, Mitsubishi Chemical Medicine Corporation, Japan) system. PCT levels were performed on an E 170 autoanalyzer (Roche Diagnostics, Tokyo, Japan) which was operated according to the electrochemiluminescence immunoassay (ECLIA) measurement principle [15]. CRP analysis was performed by a nephelometric method with BN II analyzer (Date Behring BN II, Siemens Healthcare Diagnostic Inc., Marburg, Germany) and WBC analysis was performed by Sysmex XT-2000i (Sysmex Europe GMBH) and cultures were performed by (BacT/Alert 3D; BioMerreux, Inc.) and were evaluated by the same physician. The absorbance of samples was measured at wavelength of 450 nm using a VERSA max tunable microplate reader (Molecular Devices, California, USA).

### 2.3. Statistical Analysis

Data obtained by measurements were given as mean ± one standard deviation. The main data collected for assessing patient symptoms and laboratory parameters, such as presepsin, serum PCT, CRP and WBC were compared by the Mann-Whitney* U* test. The concentrations of presepsin in SIRS patients when untreated and 48 h following treatment were compared by Student's paired* t*-test. Concentrations were expressed as median (5–95 percentile) and AUC ROC values as percent (%).

The receiver-operating characteristic (ROC) curve analyses were performed with calculation of area under the curve (AUC) for diagnosis of sepsis, severe sepsis, and septic shock during the first day of SIRS. The area under the curve (AUC) was reported to evaluate the utility of these potential markers in discriminating SIRS patients from the non-SIRS simple nephrolithiasis group. An AUC of 0.5 is considered to be no better than expected by chance, whereas a value of 1.0 signifies a perfect biomarker. Sensitivity, specificity, and cutoff were calculated in accordance with ROC curves for each biochemical marker. SPSS software (version 17.0, SPSS Inc., Chicago, IL, USA) and MedCalc (Medical Calculation Version 12.4.0, Belgium) were used for statistical analyses, and *P* < 0.05 was considered statistically significant.

## 3. Results

This study included 39 nephrolithiasis patients with SIRS in SIRS group and 25 simple nephrolithiasis patients in the non-SIRS group. The demographic parameters are listed in Table 1. There was no difference in the mean age between patients in SIRS group or non-SIRS group (*P* > 0.05) (Table 1). The analysis of the clinical data revealed stone size was not statistically different between SIRS group and non-SIRS group (*P* > 0.05), which suggested that the stone size was not an impact factor between different groups in our study. With reference to the SIRS criteria, the body temperature and blood WBC value were expressively higher in patients with SIRS as compared to those without SIRS (*P* < 0.05). In addition, urinary WBC values in the untreated patients in SIRS group were different as compared with non-SIRS group. Body temperature and WBC data were described in Table 1. The BUN and SCr levels in the SIRS group were significantly higher than those of non-SIRS group (*P* < 0.05), and the length of stay in hospital was also longer in the SIRS group (16.5 ± 7.1 d in SIRS group versus 9.4 ± 8.4 d in the non-SIRS group, *P* < 0.05) (Table 1).

**Figure 1:**
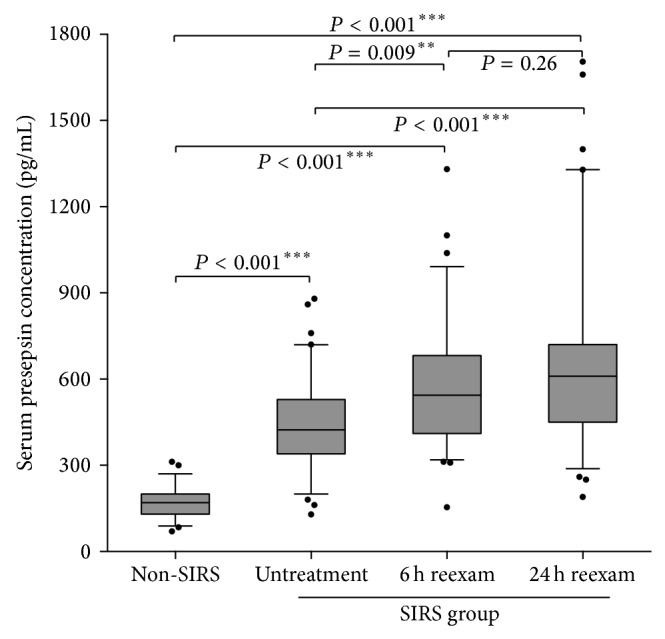
Comparison of serum levels of presepsin in different group of patients. Data are compared by Mann-Whitney* U* test. Levels are represented as ratio to the average of controls.

The presepsin concentrations were 178 (70–312) pg/mL in the non-SIRS group; 452 (129–880) ng/mL in the untreated SIRS group; 584 (154–1331) pg/mL in the SIRS group reexamination after 6 h and 660 (190–1705) pg/mL after 24 h (Table 2). The levels of presepsin were significantly higher in SIRS group compared to the non-SIRS group (*P* < 0.001). Furthermore, the levels in the SIRS group were much higher in the reexamination after 6 h and 24 h (*P* = 0.009, *P* < 0.01) (Figure 1). When presepsin was compared according to whether patients after receiving treatment or not, there was no difference between the reexamination after 6 h and after 24 h (*P* = 0.26) (Figure 1).

The serum PCT concentrations were 0.172 (0.017–1.215) ng/mL in the non-SIRS group and 0.973 (0.341–4.794) in the untreated SIRS group; PCT was 1.425 (0.698–9.176) ng/mL and 1.864 (0.712–15.392) ng/mL, respectively, in the SIRS group that was redetermined 6 h and 24 h after treatment. There were also statistically significant differences found between the non-SIRS group and the SIRS group (*P* < 0.001). Additionally, in the SIRS group the level of PCT in the reexamination was even higher than that found in the group where PCT was determined when SIRS happened (*P* < 0.001).

**Figure 2:**
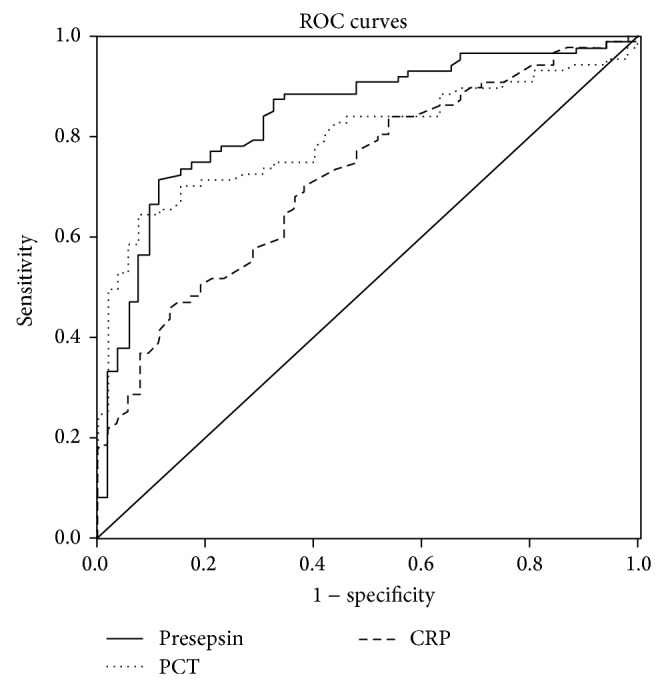
Comparison ROC curves of presepsin, PCT, and CRP in the diagnosis of SIRS in nephrolithiasis patients.

The serum CRP concentrations in the non-SIRS group, in the untreated SIRS group, and redetermined 6 h and 24 h after treatment were 6.2 (1.8–9.7), 15.9 (4.9–36.5), 28.4 (7.1–64.2), and 55.2 (13.2–82.5) mg/L, respectively. There were statistically significant differences found between the non-SIRS group and the SIRS group (*P* < 0.001) (Table 2). Timely detection results are shown in Figure 2.

**Figure 3:**
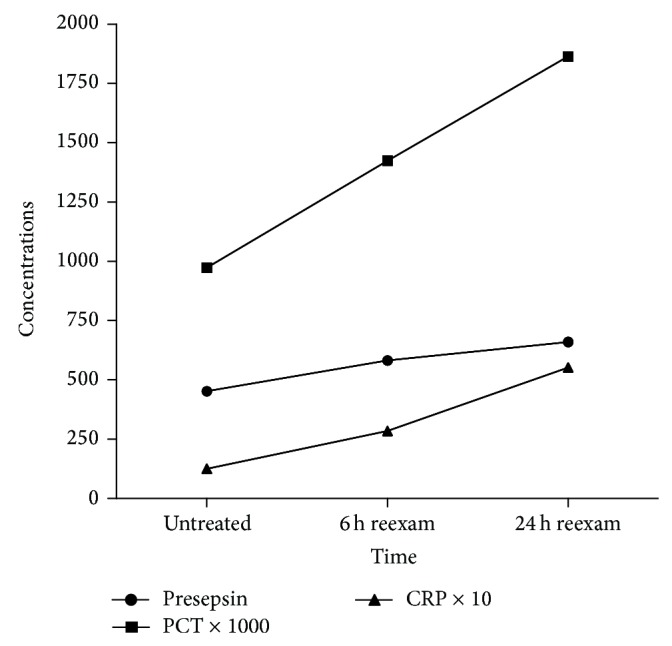
Repeat detection of three markers for SIRS in nephrolithiasis patients. Concentration axis: presepsin (pg./mL); PCT (ng/mL); CRP (mg/L).

To evaluate laboratory markers, we have used the ROC curve method wherein the areas under the curve (AUCs) for plasma presepsin, PCT, and CRP in the SIRS group were 84.6%, 79.6%, and 71.8%, respectively (Table 3). Therefore, presepsin displayed a higher sensitivity and specificity (74.7% and 88.4%) than PCT (72.6% and 85.3%) and CRP (70.7% and 75.2%). The optimal cut-off points of these three markers in the SIRS group are shown in Table 3. Daily monitorship data are shown in Figure 3.

## 4. Discussion

SIRS is an important and predictive factor for UTI, especially in urinary sepsis. However, traditional urinary tests could not efficiently reflect the progression of SIRS that had originated from the urinary tract. Mariappan et al. observed 54 patients and found that 42% of patients had a positive upper urinary tract culture (pelvic and stone), whereas only 5.6% had a positive urine culture from the bladder [7]. According to the study of Margel et al., positive detection of stone or urine culture is accepted as a relative risk for SIRS. They declared that bladder urine culture was insufficient to reveal an infection of the upper urinary tract and also reported that bigger stones are more likely to become infected than those smaller in size [21]. Similarly in our study, bacterial culture positive rates in the SIRS group were only 20.5%. In addition, our study and that of others emphasize that in the clinic, the use of microbiological methods for diagnosing SIRS is poorly sensitive and nonspecific.

Presepsin is a glycoprotein expressed on the membrane surface of monocytes and macrophages and serves as a receptor for lipopolysaccharides (LPSs) and LPS-binding proteins (LPBs). By activating a proinflammatory signaling cascade on contact with infectious agents, CD14 has a role as a recognition molecule in the innate immune response against microorganisms. During inflammation, plasma protease activity generates soluble CD14 (sCD14) fragments. One of them, called sCD14 subtype (sCD14-ST), or presepsin, is normally present in very low concentrations in the serum of healthy individuals and has been shown to be increased in response to bacterial infections [22]. Chenevier-Gobeaux et al. found a significant difference in serum presepsin levels between critically ill children with SIRS as compared with those with septic shock, even in the absence of AKI [23]. Thus, presepsin might represent a novel marker of SIRS, similar to that provided by measuring the levels of CRP and PCT.

In our study, we evaluated presepsin and found that presepsin was noticeably higher in the SIRS group as compared to the non-SIRS group (178 (70–312) and 452 (129–880) pg/mL, *P* < 0.001). This might be due to the accumulation of neutrophils within the tubular lumen during systemic inflammation and sepsis, which could lead to a noticeable increase of presepsin. Our study was concordant with other studies done with children presenting with sepsis [24]. Presepsin has been reported to be increased in AKI and chronic kidney diseases (CKD), and in our study patients presenting with CKD were excluded [25]. The mean levels of serum BUN and SCr in the SIRS group were both in the normal range, so we also excluded the involvement AKI. A previous report disclosed that peak presepsin levels were seen in the early stages of infection in animal models [26]. We aimed to assess whether presepsin would be maintained at a relatively higher level in the early stage of SIRS. We found that 6 h after the occurrence of SIRS, the concentration of presepsin was higher than that determined during active SIRS (584 (154–1331) and 452 (129–880) pg/mL, *P* < 0.001), but was not different with 24 h reexamination (660 (190–1705) and 584 (154–1331) pg/mL, *P* = 0.26). Thus, in the early stage of SIRS before the occurrence of AKI, determination of the levels of presepsin might be an objective parameter before clinical diagnosis of AKI.

PCT and CRP have been used as classic markers in the clinic for diagnosis and monitoring of SIRS during attempts to cure critically ill patients. Mokart et al. suggested that PCT could be a more reliable marker than CRP in septic patients [27]. However, another study reported that there were some limitations in using PCT as predicative marker in SIRS and sepsis, since the levels might be low or indeterminate in the early stage of the disease [28]. In our study, both PCT and CRP were significantly higher in the SIRS group as compared to the non-SIRS group. We have further evaluated the diagnostic value of PCT and CRP compared with presepsin using ROC curves analysis, whereas we found that AUCs of three markers were not different statistically (*P* > 0.05). Therefore, at the early onset of SIRS, the measurements of presepsin, PCT, and CRP were all highly sensitive and specific markers of SIRS. It is noteworthy that this is an initial study in the cohort of nephrolithiasis. However, WBC's ineffective diagnostic performance should be kept in mind.

In addition, we have valued the redetermined data 6 h and 24 h after treatment. The increase of presepsin was lower than PCT and CRP. Analysis revealed that in the early stage of SIRS, the presepsin reached the peak fast compared to PCT and CRP in the value of diagnosis. This observation has further enhanced the diagnostic importance of presepsin. In Shozushima et al.'s study, the usefulness of presepsin for diagnosis of bacterial infections was comparable to PCT, but the clinical specificity of presepsin was much higher than PCT. Higher false positive rate of PCT resulted from a subgroup of patients with secondary trauma, indicating that presepsin levels were less influenced by traumatic situations than the PCT levels [13].

In conclusion, we conclude that in our study, measurement of presepsin was a highly sensitive predictor and a useful monitoring marker in the early stage of SIRS for patients with nephrolithiasis. The ability of biomarkers, such as presepsin, to discern both the onset and resolution of SIRS will further validate use of such biomarkers in clinical diagnosis and greatly enhance our understanding of SIRS in the nephrolithiasis cohort. Further validation of serum presepsin as putative biomarkers of SIRS in this population will require a multicenter randomized study.

## Figures and Tables

**Table 1:** General information of nephrolithiasis patients with SIRS and non-SIRS.

Characteristics	SIRS group	Non-SIRS group	Total
Number of cases (*n*)	39	25	64
Gender (M/F)	24/15	16/9	40/24
Mean age (years) (mean ± SD)	39.2 ± 9.7	34.9 ± 7.1^#^	36.7 ± 8.2
Stone size (length × width × height/mm)	20 ± 4 × 8 ± 2 × 10 ± 6	15 ± 6 × 9 ± 3 × 8 ± 2	/
Body temperature (°C)	38.5 ± 0.6	36.8 ± 0.3^*^	/
Blood WBC (×10^9^/L)	12.9 ± 2.3	6 ± 1.2^*^	/
Bacteria culture positive (*N*/%)	8 (20.5%)	0^*^	/
Blood urea nitrogen (mmol/L) (mean ± SD)	7.1 ± 1.7	5.4 ± 1.2^*^	/
Serum creatinine (*μ*mol/L) (mean ± SD)	84.19 ± 7.55	67.63 ± 10.43^*^	/
Urinary WBC (/hp) (mean ± SD)	30 ± 13	4 ± 3^*^	/
Length of stay in hospital (days) (mean ± SD)	16.5 ± 7.1	9.4 ± 8.4^*^	/

^#^ *P* > 0.05; SIRS group versus non-SIRS group.

^*^ *P* < 0.05; SIRS group versus non-SIRS group.

Standard BUN 1.8–7.1 mmol/L; standard SCr 59–104 mmol/L.

**Table 2:** Laboratory markers by definitive diagnosis in SIRS and non-SIRS patients.

Laboratory findings	Non-SIRS group Median (5–95%)	SIRS group (5–95%)			*P*
		Untreated	6 h reexamination	24 h reexamination	
Presepsin (pg/mL)	178 (70–312)	452 (129–880)	584 (154–1331)	660 (190–1705)	<0.001^a,b,c,e^, 0.009^d^, 0.26^f^
PCT (ng/mL)	0.172 (0.017–1.215)	0.973 (0.341–4.794)	1.425 (0.698–9.716)	1.864 (0.712–15.392)	<0.001^a,b,c,d,e^, 0.005^f^
CRP (mg/L)	6.2 (1.8–9.7)	15.9 (4.9–36.5)	28.4 (7.1–64.2)	55.2 (13.2–82.5)	<0.001^a,b,c,d,e^, 0.003^f^

^a^Untreated in SIRS group versus non-SIRS group; ^b^6 h reexamination in SIRS group versus non-SIRS; ^c^24 h reexamination in SIRS group versus non-SIRS group; ^d^untreated versus 6 h reexamination; ^e^untreated versus 24 h reexamination; ^f^6 h reexamination versus 24 h reexamination.

**Table 3:** Diagnostic performance of biomarkers in nephrolithiasis with SIRS patients.

	Presepsin (pg/mL)	PCT (ng/mL)	CRP (mg/mL)
AUC (95% CI)	84.6 (79.8~87.1)	79.6 (75.3–82.5)	71.8 (68.7~73.9)
Cutoff	389	0.641	11.3
Sensitivity (95% CI)	74.7 (67.1–78.5)	72.6 (62.8–76.4)	70.7 (66.9–73.6)
Specificity (95% CI)	88.4 (80.2–92.6)	85.3 (82.7–89.1)	75.2 (71.5–80.2)

CI: confidence intervals.

## References

[1] Keddis M. T., Rule A. D. (2013). Nephrolithiasis and loss of kidney function. *Current Opinion in Nephrology and Hypertension*.

[2] Neisius A., Preminger G. M. (2013). Stones in 2012: epidemiology, prevention and redefining therapeutic standards. *Nature Reviews Urology*.

[3] Kreydin E. I., Eisner B. H. (2013). Risk factors for sepsis after percutaneous renal stone surgery. *Nature Reviews Urology*.

[4] Eswara J. R., Sharif-Tabrizi A., Sacco D. (2013). Positive stone culture is associated with a higher rate of sepsis after endourological procedures. *Urological*.

[5] Demirtas A., Yildirim Y. E., Sofikerim M. (2012). Comparison of infection and urosepsis rates of ciprofloxacin and ceftriaxone prophylaxis before percutaneous nephrolithotomy: a prospective and randomised study. *The Scientific World Journal*.

[6] Kumar S., Bag S., Ganesamoni R., Mandal A. K., Taneja N., Singh S. K. (2012). Risk factors for urosepsis following percutaneous nephrolithotomy: role of 1 week of nitrofurantoin in reducing the risk of urosepsis. *Urological Research*.

[7] Mariappan P., Smith G., Moussa S. A., Tolley D. A. (2006). One week of ciprofloxacin before percutaneous nephrolithotomy significantly reduces upper tract infection and urosepsis: a prospective controlled study. *BJU International*.

[8] Kibe S., Adams K., Barlow G. (2011). Diagnostic and prognostic biomarkers of sepsis in critical care. *Journal of Antimicrobial Chemotherapy*.

[9] Sridharan P., Chamberlain R. S. (2013). The efficacy of procalcitonin as a biomarker in the management of sepsis: slaying dragons or tilting at windmills?. *Surgical Infections*.

[10] Adib-Conquy M., Cavaillon J.-M. (2012). Host inflammatory and anti-inflammatory response during sepsis. *Pathologie Biologie*.

[11] Balk R. A. (2014). Systemic inflammatory response syndrome (SIRS): where did it come from and is it still relevant today?. *Virulence*.

[12] Shirakawa K., Naitou K., Hirose J., Takahashi T., Furusako S. (2011). Presepsin (sCD14-ST): development and evaluation of one-step ELISA with a new standard that is similar to the form of presepsin in septic patients. *Clinical Chemistry and Laboratory Medicine*.

[13] Shozushima T., Takahashi G., Matsumoto N., Kojika M., Okamura Y., Endo S. (2011). Usefulness of presepsin (sCD14-ST) measurements as a marker for the diagnosis and severity of sepsis that satisfied diagnostic criteria of systemic inflammatory response syndrome. *Journal of Infection and Chemotherapy*.

[14] Sugie Y., Igami K., Shoji K. (2011). Performance evaluation of the new rapid fertility assays in whole blood and plasma on PATHFAST. *Clinical Laboratory*.

[15] Okamura Y., Yokoi H. (2011). Development of a point-of-care assay system for measurement of presepsin (sCD14-ST). *Clinica Chimica Acta*.

[16] Yaegashi Y., Shirakawa K., Sato N. (2005). Evaluation of a newly identified soluble CD14 subtype as a marker for sepsis. *Journal of Infection and Chemotherapy*.

[17] Glück T., Silver J., Epstein M., Cao P., Farber B., Goyert S. M. (2001). Parameters influencing membrane CD14 expression and soluble CD14 levels in sepsis. *European Journal of Medical Research*.

[18] Lucarelli G., Mancini V., Galleggiante V. (2014). Emerging urinary markers of renal injury in obstructive nephropathy. *BioMed Research International*.

[19] Bone R. C., Balk R. A., Cerra F. B. (2009). Definitions for sepsis and organ failure and guidelines for the use of innovative therapies in sepsis. The ACCP/SCCM Consensus Conference Committee. American College of Chest Physicians/Society of Critical Care Medicine. 1992. *Chest*.

[20] Mehta R. L., Kellum J. A., Shah S. V. (2007). Acute Kidney Injury Network: report of an initiative to improve outcomes in acute kidney injury. *Critical Care*.

[21] Margel D., Ehrlich Y., Brown N., Lask D., Livne P. M., Lifshitz D. A. (2006). Clinical implication of routine stone culture in percutaneous nephrolithotomy—a prospective study. *Urology*.

[22] Ulla M., Pizzolato E., Lucchiari M. (2013). Diagnostic and prognostic value of presepsin in the management of sepsis in the emergency department: a multicenter prospective study. *Critical Care*.

[23] Chenevier-Gobeaux C., Trabattoni E., Roelens M., Borderie D., Claessens Y.-E. (2014). Presepsin (sCD14-ST) in emergency department: the need for adapted threshold values?. *Clinica Chimica Acta*.

[24] Dessì A., Corsello G., Stronati M. (2014). New diagnostic possibilities in systemic neonatal infections: metabolomics. *Early Human Development*.

[25] Palmiere C., Augsburger M. (2014). Markers for sepsis diagnosis in the forensic setting: state of the art. *Croatian Medical Journal*.

[26] Sandquist M., Wong H. R. (2014). Biomarkers of sepsis and their potential value in diagnosis, prognosis and treatment. *Expert Review of Clinical Immunology*.

[27] Mokart D., Merlin M., Sannini A. (2005). Procalcitonin, interleukin 6 and systemic inflammatory response syndrome (SIRS): early markers of postoperative sepsis after major surgery. *British Journal of Anaesthesia*.

[28] Becker K. L., Snider R., Nylen E. S. (2008). Procalcitonin assay in systemic inflammation, infection, and sepsis: clinical utility and limitations. *Critical Care Medicine*.

